# Attitudes of physicians and patients towards disclosure of genetic information to spouse and first-degree relatives: a case study from Turkey

**DOI:** 10.1186/1472-6939-15-39

**Published:** 2014-05-16

**Authors:** Aslihan Akpinar, Nermin Ersoy

**Affiliations:** 1 Department of History of Medicine and Ethics, Hacettepe University Faculty of Medicine, Ankara, Turkey; 2 Department of History of Medicine and Ethics, Kocaeli University Faculty of Medicine, Kocaeli, Turkey

## Abstract

**Background:**

When considering the principle of medical confidentiality, disclosure of genetic information constitutes a special case because of the impact that this information can have on the health and the lives of relatives. The aim of this study is to explore the attitudes of Turkish physicians and patients about sharing information obtained from genetic tests.

**Methods:**

The study was carried out in Kocaeli, Turkey. Participants were either paediatricians and gynaecologists registered in Kocaeli, or patients coming to the genetic diagnosis centre for karyotype analysis in 2008. A self-administered paper questionnaire was given to the physicians, and face-to-face structured interviews were conducted with patients. We used a case study involving a man who was found to be a balanced chromosome carrier as a result of a test conducted after his first baby was born with Down's syndrome. However, he refused to share this information with his wife or his siblings. Percentages of characteristics and preferences of the participants were calculated, and the results were analysed using Kruskal-Wallis test.

**Results:**

A total of 155 physicians (68% response rate) and 104 patients (46% response rate) were participated in the study. Twenty-six percent of physicians and 49% of patients believed that genetic information belongs to the whole family. When participants were asked with whom genetic information should be shared for the case study, most of the physicians and patients thought the physician should inform the spouse (79%, 85%, respectively). They were less likely to support a physician informing a sibling (41%, 53%, respectively); whereas, many thought the testee has an obligation to inform siblings (70%, 94%, respectively).

**Conclusions:**

Although Turkey’s national regulations certainly protect the right of privacy of the testee, the participants in our study appear to believe that informing the spouse, who is not personally at risk of serious damage, is the physician’s responsibility, while informing siblings, is the testee’s responsibility. Therefore we believe that opening ethical discussions with clinicians about the sharing of genetic information, establishing guidelines for practice and sharing these guidelines and the reasons behind them with the wider population, will help to pre-empt ethical dilemmas.

## Background

When considering the principle of medical confidentiality, it is argued that disclosure of genetic information is a special case because of the impact that this information can have on the health and lives of relatives [1,2]. This claim triggers discussions about exceptions to the principle of medical privacy (as outlined within the context of psychiatric care and the care of people with HIV/AIDS [3]) in relation to genetic information. Is it possible to apply the previously established condition that disclosure of information is permitted if there is ‘a clear and present danger’ which can only be avoided by this disclosure [4], to the sharing of genetic information? Or should we establish new rules for genetic information which enable an individual to make informed choices whether or not the damage can be prevented?

The answer to these questions will be determined by whether we take the Enlightenment-rooted individual of Anglo-Saxon culture or the family as the unit of privacy [5]. For example, while the individual's privacy is prioritized in the USA where liberal individualist ethics is dominant, there is an entirely different perspective on sharing genetic information in Japan, where Confucian ethics are dominant and family relationships more central [6]. Even within Western societies, there are different views about the ethics of sharing genetic information, all of which may be reasonable and well thought-out. For example, while Australia and Israel accept the disclosure of genetic information even in the cases in which the risk is not clear, including cases involving unborn babies, the consent of the patient is an absolute pre-requisite in Turkey and France [7-9].

These differences could risk opening the door to ethical relativism, and responding to these conflicts involves a re-examination of principlism and openness to the possibility of applying alternative ethical approaches, other than liberal individualism [10]. It is now beginning to appear that, for the time being at least, the traditional medical targets of treatment and cure have been superseded by targets of diagnosis and prevention which rely heavily on the free availability of medical genetic information. This shift in emphasis has resulted in a redefinition of what constitutes ‘benefit’ and what constitutes ‘harm’ where genetic information is concerned. These revised, and culture-specific, definitions of benefit and harm in the context of disclosure of genetic information [11] may contribute to the drawing up of new, more comprehensive rules and guidelines for treating sensitive genetic information.

This new approach argues that the opinion of a majority of both directly and indirectly affected individuals will be crucial in establishing the rules governing the disclosure of genetic information. For example, although the most common model of the family in Turkey is no longer an extended family group but rather a modern, nuclear group [12], a quarter of all Turkish marriages are still consanguineous, mainly between first cousins [13]. In addition to this, although rapid social and cultural change has lessened the importance of the family as a unit, and has hastened the decline of group consciousness and loyalty within the family group, while the blurring of gender roles has also changed the dynamics, the family unit is still the most important primary and intimate unit within Turkey [14]. The resulting family structure, with its high proportion of marriages between cousins, increases the likelihood of babies being born with genetic defects unless genetic information is shared in a responsible manner.

Cytogenetic or molecular genetic tests are carried out in most Turkish medical laboratories and departments of medical genetics within medical faculties. They are also carried out in the increasing number of private medical laboratories. In certain cases, the difficulties experienced in providing genetic counselling, where the aim is to give information about the importance and possible outcomes of genetic tests, provide a context for a number of ethical problems. The absence of any national standards for the use of genetic information makes the field fraught with difficulties.

The aim of this study is to explore the attitudes of related parties to the issue of genetic information and how it should be shared. This exploration is considered with particular reference to a recent case which took place in Turkey, and involves subjecting this case to an ethical analysis using existing ethical rules and results from the other countries.

## Methods

### Sample and setting

This study was ^a^carried out in Kocaeli, the second largest industrial province in Turkey after Istanbul.

The study was carried out in two phases. The first phase focused on establishing which areas of specialization most frequently demand a genetic test. To determine this, we reviewed annual statistics (in 2008) of the Medical Biology and Genetics Laboratory - in which molecular genetics and cytogenetic testing are performed – located within the Faculty of Medicine of Kocaeli University^b^. It was discovered that the departments in which genetic testing were most likely to be requested were (1) obstetrics and gynaecology, and (2) paediatrics. Professionals specialising in these fields in the Kocaeli University Hospital were contacted and asked for write-ups of the cases where they had experienced an ethical dilemma. These case studies were narrated by the researchers in a manner which brought out the ethical dimension, and this format was finalized between the researchers and specialists in a face-to-face meeting.

The Case extracted from this process and used in this study was written up by a neonatologist. It concerns a person named Mahmut, (All personal characteristics and names were fictional in this case) who was found to be a balanced chromosome carrier as a result of a test conducted after his first baby was born with Down's syndrome. However, he refused to share this information with his wife or his siblings. In a balanced translocation, “pieces of chromosomes are rearranged but no genetic material is gained or lost in the cell. The individual with a ‘balanced’ translocation will usually have the correct amount of genetic information for normal development. But there is an increased chance that there will be reproductive consequences due to the child receiving an ‘unbalanced chromosome complement’ – i.e., the child has more or less chromosomal material than usual [15].”

The second phase, a cross-sectional research study, was performed in Kocaeli. Two categories of people were invited to participate: (1) all specialists working in the fields of obstetrics and gynaecology or paediatrics and serving in Kocaeli; and (2) the patients who are referred to the Laboratory of Medical Biology and Genetics in the Faculty of Medicine by physicians working in related departments and who gave their consent for genetic tests. Structured questionnaires, developed by the researchers, were left for the physicians to fill in, while the patients and parents were given structured face-to-face interviews covering the same series of questions. Patients and parents were informed about the study, and emphasising voluntary participation, oral informed consent was obtained. The physicians were reminded that returning a completed study form implies informed consent. This study was approved by the Human Research Ethics Committee of Kocaeli University (Decision Number: 2008-İAEK3/12).

### Questionnaire and data analysis

The questionnaires for the study were developed in the following way. Firstly, ethical issues arising from genetic testing were identified through a literature search. These issues were then used to gather opinions from medical ethics specialists, medical biology and genetics specialists, gynecologists and obstetricians and child health and disease experts, in order to create a draft questionnaire. The draft questionnaires were shaped based on the characteristics of the two groups that would be targeted by the study and were checked by an expert in the Turkish language. Pilot studies were conducted involving two groups, one of ten physicians and the other of ten patients to ensure clarity of the questionnaires (Additional files 1, 2, 3 and 4).

In this main study the questions which explored the socio-demographic and professional characteristics of the groups and the attitudes of these groups regarding the ownership of genetic information both in general and with specific reference to the case study were used. The first four statements about the case were related to the ethical obligations of the physician, the fifth statement was related to Mahmut's personal responsibility and the last statement concerned the obligations of the state in terms of social justice. The relation between the answers of the parties to the questions and the personal characteristics of the participants and the professional features of the physicians, and the relation between the information level of the patients/parents and the attitudes of the parties towards the issue of ownership of genetic information were analyzed with the *Kruskal-Wallis* test and p < 0.05 was accepted as the significance level.

## Results

### The case

26-year-old Nurgül gave birth to a premature baby. The baby had hypertelorism, simian crease, endocardial cushion defects, chronic lung disease and pulmonary hypertension. A chromosome anomaly was detected in the test which had been suggested by Dr. Elif, the neonatalogist, who suspected that the baby might have Down syndrome. The mother, who was hoping to have another baby in the future, insisted that the cause of the Down syndrome be investigated and asked for tests to be carried out both on her and on her husband. Realizing that the father was unwilling to participate, the neonatalogist Dr. Elif gave information to the spouses about prenatal diagnostic tests which could be carried out in the course of a future pregnancy. Two weeks later, Nurgül and her husband Mahmut, who could not withstand his wife’s determination, applied to have the test. Mahmut was identified by the test results as a balanced translocation carrier. Mahmut has younger siblings, all of whom may wish to have children in the future. Dr. Elif suggested to Mahmut that it would be helpful to them if he were to share this information about his condition with his first degree relatives and his wife who was keen to start a second pregnancy. However, Mahmut said that his communication with his relatives was not at all good anyway, and he was not planning to have a second baby. For these reasons, he refused to tell his family members, or his wife, about the test result.

Of the physicians who were approached to take part in the research, 155 (68%) participated. The age range of this group was 30–60 years, with a mean age of 44.4 ± 10.3. Socio-demographic characteristics of the participating physicians are shown in Table 1.

**Table 1:** Personal and professional characteristics of the physicians (N = 155)

**Personal characteristics**	**n (%)**
**Gender**	
Male	90 (58.1)
Female	65 (41.9)
**Age**	
30s	57 (36.8)
40s	56 (36.1)
50s and over	42 (27.1)
**Children**	
Has child/children	131 (84.5)
No children	24 (15.5)
**Area of Specialty**	
Paediatrics	82 (53.0)
Gynaecology and Obstetrics	73 (47.0)
**Years in the Field**	
≤ 10 years	72 (46.5)
>10 years	83 (53.5)
**Information level about genetic testing**	
Sufficient	(32.3)
Medium	(48.4)
Not sufficient	(19.4)
No knowledge	-

Of the 155 participating physicians, 80.6% (n = 125) stated that they suggest genetic testing to their patients as part of their daily practice, while 40% of the physicians (n = 50) stated that they suggest genetic testing to their patients at least once a month.

When the physicians were asked what they thought about the ownership of genetic information, 62% of them stated that it belongs to the individual, 26% of them said it belongs to the family and 12% of them said that it belongs to humanity.

The average of age of the 104 patients/parents (46% response rate) participating in the research was 32.4 ± 7.0 (18-47). As all participants (whose characteristics are shown in Table 2) had been pre-diagnosed, they had all already given blood for karyotype analysis either on their own or behalf of that of their children. Nearly two thirds – 64.4% (n = 67) – of the participants had been referred to the genetics laboratory from the department of obstetrics and gynaecology, and the remaining 35.6% (n = 37) had been referred by the department of paediatrics. Only 58% of these participants said that they had been given sufficient information about the test. When the patients were asked about the ownership of the genetic information; 39% stated that it belongs to the individual, 49% to the family and 12% to humanity.

**Table 2:** Personal characteristics of those undergoing genetic testing (N = 104)

**Demographical features**	**n (%)**
**Gender**	
Male	46 (44.2)
Female	58 (55.8)
**Age**	
Between 18 – 29	35 (33.7)
30s	53 (51.0)
40s	16 (15.4)
**Children**	
Has child/children	48 (46.2)
No children	56 (53.8)
**Level of education**	
≤8 years	52 (50.0)
9-11 years	31 (29.8)
≤12 years	21 (20.2)
**Information level about the genetic tests**	
Sufficient	-
Medium	22 (21.2)
Not sufficient	36 (34.6)
No knowledge	46 (44.2)

The responses of the participants to the case study, and the relationship of these responses to the independent variable are shown in Table 3.

**Table 3:** The relation between the responses to the statements in the case and the personal and/or professional characteristics of the parties

**Statements**		**Physicians n (%)**	**Patients n (%)**
*The physician should respect Mahmut's decision*	I agree	39 (25.8)	13 (12.5)
	Neutral	24 (15.9)	7 (6.7)
	I disagree	88 (58.3)	84 (80.8)
*Significance (N = 151; N = 104)*		*NS*	*Gender, p = .013*
*The physician should inform the spouse*	I agree	117 (78.5)	88 (84.6)
	Neutral	16 (10.7)	4 (3.8)
	I disagree	16 (10.7)	12 (11.5)
*Significance (N = 149; N = 104)*		*Age, p = .037; Level of information, p = .024*	*NS*
*The physician should avoid revealing the truth to the spouse to protect the unity of the family.*	I agree	15 (10.1)	10 (9.6)
	Neutral	21 (14.1)	2 (1.9)
	I disagree	113 (75.8)	92 (88.5)
*Significance (N = 149; N = 104)*		*NS*	*Gender, p = .005*
*The physician should inform the siblings*	I agree	61 (41.2)	55 (52.9)
	Neutral	43 (29.1)	5 (4.8)
	I disagree	44 (29.7)	44 (42.3)
*Significance (N = 148; N = 104)*	*Age, p = .007; Years in department, p = .007; Ownership, p = .002;*		*Ownership, p = .025*
*Mahmut has an obligation to inform his siblings and to advise them to take test*	I agree	106 (70.2)	98 (94.2)
	Neutral	26 (17.2)	3 (2.9)
	I disagree	19 (12.6)	3 (2.9)
*Significance (N = 151; N = 104)*		*Ownership, p = .025*	*NS*

A small proportion of both groups agreed with the statement that *Dr. Elif should respect Mahmut’s decision*. Men were more likely to respect Mahmut's decision than women in the group of patients (Table 3).

The majority of both groups agreed with the statement that *Dr. Elif should notify Mahmut’s wife about the results* despite Mahmut’s request to keep his genetic information confidential. Physicians who were over fifty years old and believed their knowledge about genetic testing to be ‘sufficient’ were the most likely group to agree with the statement about notifying Mahmut’s wife (Table 3).

The majority of participants disagreed with the statement that *Dr. Elif should avoid revealing the truth to Mahmut’s wife in the interests of family unity*; however men in the patient group were more likely than women in the same group to agree with this statement.

The statement *Dr. Elif has a responsibility to warn Mahmut’s siblings about the genetic test results* provoked a mixed reaction with agreement from 41% of the physicians and 53% of the patient group. The older and more experienced the physicians were, the more likely they were to agree about the importance of warning Mahmut’s siblings. In addition, the idea that it was the physician’s responsibility to inform siblings was one that found particular support from physicians and from those patients who think genetic information belongs to the family.

The majority of the participants agreed with the statement that *Mahmut has an obligation to reveal the truth to his siblings and to advise them to take test*. Most of the physicians who believe genetic information belongs to humanity agreed with the idea that the responsibility of warning siblings belongs with Mahmut himself.

The last question about this case was *whether there was an obligation for the state to provide free genetic testing for people* in the case of chromosomal abnormalities which affect subsequent generations. The response to this was a positive one with 78% of the physicians and 94% of the patients agreeing with the idea that the state should be obliged to make such tests available free of charge. No relationship was detected between these responses and the independent variables. Those patients who disagreed with this idea thought that the state should only pay for testing those people who could not afford to pay for the tests themselves.

### Limitations

There were several limitations to this study. First, since we conducted the study only in Kocaeli, the findings cannot be generalised to the Turkish population as a whole. Second, there is potential for selection bias as the participants were those who were willing to give their time to fill in the questionnaire. Third, the opinions expressed by the participants may not accurately reflect what would happen in a real world situation, rather than a hypothetical exercise.

## Discussion

The extent of the individual’s right to privacy, and the extent to which the physician has an obligation to protect the third parties involved, constitute the ethical dilemma at the heart of the case-study. -One quarter- of the physicians surveyed and -one eighth- of the patients believed that Mahmut had a right to expect this information to be kept confidential. The majority of those who supported this view were male patients. The responses of participants to questions about the issue of protecting the unity of the family overlapped with the responses about the respect for the privacy.

### Effect of gender on disclosure of third parties

Gender has a clear effect on attitudes towards concealing this information, both in our study and in a similar research study carried out in France. In both studies, a substantial proportion of the men surveyed were sympathetic to the idea that test results should not be disclosed if the subject of the tests so wished [16]. Although it could be argued that this sympathy on the part of males is likely to be affected by the patient’s gender, this result is also consistent with other research findings which show that women are more likely to take responsibility for warning others who might also be at risk [17,18]. When considered from the perspective of feminist ethics, this finding seems to support the idea that it is characteristic of women to protect everyone's benefit, take care of other people's needs and sympathize with others [19]. For example, it has been reported in a Canadian study that, among the individuals who have BRCA1/2 mutation, women are more willing to share the information about risk by contacting others, even distant relatives [20].

### Medical confidentiality versus protecting others

The obligation of protecting patient confidentiality, which is also a requirement of respecting patient autonomy and privacy, is an integral part of the duty of medical confidentiality – which itself is one of the earliest obligations of medicine. The main exception to this duty arises when third parties might be exposed to an unacceptable degree of damage, especially within the context of HIV/AIDS or psychiatry [4]. In Turkey there are no legal standards about a duty to warn in such situations. Although there is a bill of law about HIV/AIDS in which partner notification in limited situations is mentioned, it is not yet legalized [21]. There are also public health reporting requirements for some contagious diseases; however, these notifications are only required for establishing health policies and do not include partner notification [22]. On the other hand, both physicians and those who have had genetic tests seem to find the idea of sharing the test results with a spouse more appealing than the idea of sharing them with another individual, such as a sibling, who runs the risk of experiencing physical or social damage on the basis of these genetic test findings. This situation, which cannot be evaluated within the scope of the available exemptions of the medical confidentiality, is noteworthy. Although the carriers of balanced chromosome will not have a risk of experiencing a serious medical problem themselves, it is possible that their children will. A definite decision needs to be made, therefore, about whether this risk is sufficient to allow violation to the principle of medical confidentiality [16,23], because concealment of a secret such as that involving Mahmut may cause harm and considerable distress to any third parties who may be affected.

There is a conflict here between the obligations of the physician in terms of medical confidentiality and the ethical obligation to prevent other people from incurring damage or minimizing the damage. There is also a conflict between the principles of nonmaleficience and justice [3]. Although Mahmut’s request for his test result not to be disclosed to his wife was not met with approval from the participants in our study, there is only one article in the *Regulation on the Centres for Diagnosis of Genetic Diseases* (the only regulation produced on ethical issues related genetics within Turkey) covering this topic. The article states that "…genetic test results cannot be revealed to third parties without the consent of the person [9]”. This ethical approach is supported by the *Regulation of Patients’ Rights*[24] and the Turkish Medical Association’s (TTB) *Code of Medical Ethics*[25] within the scope of the obligation to respect patient privacy and confidentiality. The only exemption stated in the TTB’s *Code of Medical Ethics* is when the life itself is endangered: "The obligation of confidentiality of the physician becomes invalid in circumstances where keeping confidentiality would put the life of the patient or other people in jeopardy (article 9)". The exemptions in which the breach of the confidentiality can be approved ethically are grounded in the possibility and degree of damage in these ethical codes.

While the current regulations include this proviso, an ethical concern arises from the fact that physicians (in common with the patients) prefer to disclose the genetic information to the patient’s wife rather than to his siblings. Older, more experienced physicians – who presumably have more knowledge about the implications of genetic testing – share this concern. The duty of the physician regarding the prevention of damage to third parties is likely to make them more aware about people who are at serious risk of damage. Therefore, the benefit and harm of revealing or hiding the information should be evaluated in each case with respect to the principle of proportionality [23,26]. When the ratio of benefit and harm resulting from sharing the information with the spouse is evaluated objectively, the fact that the mother who gives birth to a baby with Down syndrome is already at higher risk than other mothers in her next pregnancies [27], makes it difficult to justify violating Mahmut’s confidentiality when considering the ‘clear and present danger’ rule. Nevertheless, the physician is expected to encourage Mahmut to discuss his results with his wife Nurgül so that she can make informed decisions about future pregnancies and have choices about whether to give birth to a baby with Down syndrome. This will also increase honesty within the marriage. In addition, the physician should encourage Nurgül to speak about her own health condition, and her plans about having children, with her husband [5].

The patients interviewed felt less responsible for warning siblings who might be at risk of being damaged by these results than they did about warning the spouses. However, physicians who were in their fifties and who were more experienced were more likely to give serious consideration to the potential harm to other people. The belief that the test results belong to the family as a whole appears to be behind the willingness to disclose information to siblings rather than to the spouse. Our opinion is that the number of participants in our study who felt that the primary responsibility for informing siblings lay with Mahmut rather than with the physician supports this theory (Table 3). Although some of the patients appear to hold the view that it should be Mahmut who should inform his siblings about the result of the genetic testing, rather than the physician, because it would be difficult for physicians to communicate with the patient’s relatives in their intense work environment, the physicians who believe that genetic information belongs to the family as a whole are even more likely to support this course of action (Table 3).

Research studies carried out with groups of balanced chromosome carriers in the USA [28] and England [29] found that brothers, sisters and other relatives were likely to be given information, in contrast to the studies carried out in Germany [30] and France [16]. In these latter countries, as in our case, the patients being tested refused to inform their relatives. The reasons they gave for their decision were that they want to take decisions about their family planning with reference to the present situation rather than to the future [16]; psychological reactions like guilt and shame; the fear of being stigmatized in the family on account of being a carrier; feeling inadequate to the task of informing relatives, or denial of the results and their implications and subsequent depression [30]. All of the participants in a Canadian study on patients with breast cancer felt not only that they had a responsibility to share the information but also that their relatives have the right to know [20].

It is important to determine the degree of harm that can be caused by the failure to share information, and seek reliable and valid evidence about whether the sharing of information can help prevent harm before making a decision about genetic information which could violate the principle of medical confidentiality [23,26,31]. Although the degree of potential risk of harm may change with time, as the field of medical genetics advances, the findings currently available indicate that the likelihood of adults appearing phenotypically normal but carrying balanced chromosome anomaly is 1/500 in the general population, and 80% of these cases are hereditary [29,32]. Consequently, it is possible to discuss certain risks, such as having a baby with an unbalanced chromosome anomaly (10%-15%) [30,32], spontaneous miscarriage (25%-50%), infertility, or recurrent miscarriage in relatives. However, these risks can be prevented with prenatal or preimplantation cytogenetic diagnostic tests [30,33,34]. As a result it is argued that, when genetic risk and diagnosis is taken into consideration, sharing the information that a person is a carrier of balanced chromosome anomaly, even with distant relatives, can be justified [30]. On the other hand, researches in France and the USA suggest that some relatives who are informed by the testees do not feel any need to find out more information [16,30]. The suggestion that testees cannot inform their relatives effectively [35], or that the information can be misinterpreted [16], makes it all the more important that physicians provide relevant knowledge to third parties in the interest of preventing harm.

The widely accepted idea that the individual has a moral obligation to inform his/her relatives [11,17,20,23,36] was evidently also held by the patients in our research. This result can be discussed within the framework of the individual's responsibility to his/her relatives. For example, as mentioned by Raz and Schicktanz [37], according to Kenen (1994) and Hallowel (1999) the increase in access to genetic information also increases the onus on the individual concerned to share his/her genetic information with any relatives who might also be affected, and Konrad (2003) defines the conflict between the imperative to disclose and the desire to hide the information as a moral conflict. Most of the German subjects affected by a genetic disorder believe they have a duty to warn their relatives, especially when preventive actions can be taken [37]. Informing the people at risk is also considered less important in our research and in a study conducted in Australia [11].

On the other hand, those who believe that genetic information belongs to the family as a whole tend to support the disclosure of such information, coinciding with the communitarian ethical approach which pursues the benefit of all family members who might be affected by the problems identified [38,39]. In addition to this, the virtues expected from Mahmut – such as honesty in his sharing of information with his wife, and altruism in sharing the truth with his siblings – may provide guidelines for ethical behaviour [40].

### Confidentiality and insurance coverage of genetic testing

The responsibilities of the state, in terms of enabling access to genetic tests and minimizing the damage people might experience if they lack access to such tests should be discussed with reference to social justice. The state's obligation relating to equal allocation of medical resources among the people who have similar needs is based on social justice. The responsibility of the state to enable the access to genetic tests not only for those who can pay but also for those who need them but who cannot pay [41], is based on an understanding that it is important to prevent damage, as far as possible, in those who are most likely to suffer from this damage [42].

The physicians and the patients in our study believed that, in situations where the reproductive choices for healthy generations might be affected, the state has an obligation to provide the relevant genetic tests free of charge. The regulations in Turkey appear to be compatible with these expectations. Although genetic tests (prenatal, cytogenetic and molecular genetic) are covered by health insurance based on medical need, these regulations also allow people who do not have health insurance to benefit from these expensive tests [43]. However, physicians and patients in our study believe that the cost of the tests should be met by the state even if there is no medical indication, as in our case. In fact, the state policy with regard to genetic tests/advanced diagnostic tests is determined by a cost and benefit analysis of genetic screening programs. The cost-benefit analysis for these programs is measured by their effectiveness in decreasing the incident and mortality/morbidity of the relevant genetic disease [44]. For example, the newborn screening program in Turkey does not include a test for hemoglobinopathy because such a program would have a cost-benefit advantage only if the national incidence of this condition was high. In Turkey, it is low, and so premarital blood tests are considered sufficient [45].

The regulations relating to the coverage of DNA tests, other than the judicial and medical indications, by health insurance [46], provide a legal framework in which the medical profession and the state can work together for the common good. The evidence-based medical indication, which should be pursued by the physician in the decision-making process, is relied upon within the context of the genetic tests, and as long as there is medical indication, the test fees are covered by health insurance [46]. ^c^As a result, this regulation supports the additional duty of the medical profession, which includes the fair allocation of medical resources and prevention of their unnecessary use in terms of the principle of justice, along with the duty to provide medical treatment [47]. As observed in this case study, when there is doubt about the material benefit of the test, it seems difficult to justify the test and deem it a fair allocation of medical resources.

## Conclusions

The family unit is still the most important unit among primary and intimate groups in Turkey [14]. The frequency of consanguineous marriage in Turkey may both increase the number of the babies born with genetic disorders, and give rise to more conflicts over whether or not to share genetic information.

In our case study, which is valuable in terms of demonstration of the importance of cultural differences, the following conclusions were reached:

● Although many physicians believe that genetic information belongs to the individual, which suggests they support individual privacy, they also believe that, in certain situations, it is justifiable to breach the confidentiality of a testee.

● The majority of the patients interviewed appear to believe that informing the spouse, who is not personally at risk of serious damage at present, is the physician’s responsibility, while informing siblings, who have the possibility of facing risks, is the testee’s responsibility. This tendency to believe the spouse should be informed by the physician may arise from the importance given to the institution of the family, and the perception of the family as the keystone of society in Turkey.

● Finally, the majority of those patients interviewed believe that it is the responsibility of the state to provide genetic testing free of charge, even in situations where medical indication of the genetic test cannot be presented clearly and cannot be associated with just allocation of resources. The Turkish Department of Social Security Institution prefers to base such decisions upon the clarity of the evidence that can be gathered and on its applications. On the other hand, this does not seem to be an entirely satisfactory solution because of the concerns for possible discrimination among patients in the future.

All these results demonstrate the necessity and the importance of informing all parties about the availability of genetic counselling and testing. Such information has not as yet been disseminated sufficiently in Turkey. In addition, we believe that opening ethical discussions with clinicians about the sharing of genetic information, establishing guidelines for practice and sharing these guidelines and the reasons behind them, with the wider population will help to pre-empt ethical dilemmas. As outlined in the case study, participants prefer breaching confidentiality in situations like those described the case story. Although we do not know the reasons underlying this belief, it is noteworthy and should be considered when establishing ethical guidelines for exceptions to medical confidentiality in Turkey.

## Endnotes

^a^This project, of which only a part is presented here, is Aslihan Akpinar’s doctoral dissertation titled "Ethics in Using Genetic Information: Attitudes and Preferences of Physicians and Testees".

^b^The Hospital of Kocaeli University is the oldest research hospital serving the West of the Black Sea Region and is a center for specialist referrals from the services.

^c^However, the Turkish Department of Social Security demands the original document containing the test result before reimbursing costs. This rule, which was originally instituted to prevent the unnecessary use of the genetic tests, should be revised on the basis of potential damage to the individual.

## Competing interests

We certify that we have no competing interests in respect of this manuscript.

## Authors’ contributions

AA and NE designed the study and developed the questionnaires. AA did data collection, statistical analysis, results interpretation and writing up the manuscript. NE critically evaluated the results and the manuscript. AA was a research assistant at Kocaeli University when the study was conducted. Both authors read and approved the final manuscript.

## Pre-publication history

The pre-publication history for this paper can be accessed here:

http://www.biomedcentral.com/1472-6939/15/39/prepub

## Supplementary Material

Additional file 1 Physician’s Form in Turkish. Click here for file

Additional file 2 Physician’s Form in English. Click here for file

Additional file 3 Patient’s Form in Turkish. Click here for file

Additional file 4 Patient’s Form in English. Click here for file
