# Supplementary material for: Attitudes of physicians and patients towards disclosure of genetic information to spouse and first-degree relatives: a case study from Turkey
Source: BMC Med Ethics. 2014 May 16;15:39. doi: 10.1186/1472-6939-15-39 (PMC4029893; doi:10.1186/1472-6939-15-39)
Supplement: Additional file 1 — Physician’s Form in Turkish. [file 1472-6939-15-39-S1.pdf]

Genetik Bilginin Kullanılmasında Etik: Tarafların Tutum ve Tercihleri - *Hekim Formu*

Değerli Katılımcı,

1990'ların sonlarından bu yana insan genetiği alanında olağanüstü gelişmeler yaşanmaktadır. Klinik genetikte ve özel olarak genetik testlerinde yaşanan bu gelişmeler özerklik, bireysel gizlilik ve mahremiyet gibi konulara ilişkin etik sorunların da ortaya çıkmasına neden olmaktadır. Elde edilen bireysel <u>genetik bilgilere</u> kimlerin, hangi şartlarda ulaşabileceği hakkında önemli oranda etik tartışmalar başlamıştır. Planladığımız bu çalışmayla, hekimlerden ve test yaptırmış kişilerden, elde edilen bireysel genetik bilgilerin açıklanması ve paylaşılması hakkında tutum ve tercihlerini öğrenmek amaçlanmıştır. Tarafların görüşleri doğrultusunda, yerel ve ulusal alanda bireysel genetik bilgilerin açıklanması ve gizliliğinin korunması hakkında bir etik tartışma başlatabilmeyi ve bu bağlamda kendimize özgü kuralların oluşumu için öneriler sunabilmeyi hedeflemekteyiz. ***Katkılarınız için teşekkür ederiz.***

Prof. Dr. Nermin ERSOY (Tez Danışmanı), Arş. Gör. Aslıhan AKPINAR (Tez Yürütücüsü)

I. KİŞİSEL ÖZELLİKLERİNİZ

1. **Cinsiyetiniz :** ☐ Erkek ☐ Kadın **2.** **Yaşınız:........** **3.** **Çocuğunuz:** ☐ Var ☐ Yok

4. **Uzmanlık alanınız ve varsa yan dal uzmanlık alanınız? …………………………………**

5. **<u>Uzman olarak</u> kaç yıldır görev yapıyorsunuz? ……………**

6. **Çalıştığınız kurumun türü** (İlgili seçeneklerden birden fazla işaretleyebilirsiniz)**:**

☐ Devlet Hastanesi ☐ Üniversite Hastanesi ☐ Özel Hastane ☐ Muayenehane

7. **Genetik testler hakkında ne kadar bilgi sahibisiniz?**

☐ Yeterli ☐ Orta düzeyde ☐ Yetersiz

8. **Dinin yaşamınızdaki yeri?** ☐ Önemlidir ☐ Ne önemli ne önemsizdir ☐ Önemli değildir

9. **Politik görüşünüzü tek kelimeyle nasıl ifade edersiniz? ………………**

II. GENETİK TESTLERE İLİŞKİN UYGULAMALARINIZ

10. **Günlük uygulamalarınız sırasında hastalarınıza genetik test öneriyor musunuz?**

☐ Evet ☐ Hayır (<u>Hayır ise</u> lütfen 15. soruya geçiniz ➷)

11. **Hangi sıklıkla genetik test öneriyorsunuz?**

☐ Haftada en az 1 kez ☐ Ayda en az 1 kez ☐ 6 ayda en az 1 kez ☐ ya da (*lütfen yazınız*) ………………

12. **Önerdiğiniz genetik testlere ilişkin bilgileri nereden edindiniz?**

☐ Tıp eğitimimden ☐ Uzmanlık eğitimimden ☐ İzlediğim bilimsel yayınlardan

☐ Aldığım kurs/seminerlerden ☐ ya da (*lütfen yazınız*) ……………………….

13. **Hastalarınıza genetik test sonuçları hakkında hangi durumlarda bilgi veriyorsunuz?**

☐ <u>Her zaman</u> hastalarıma sonuç hakkında bilgi veriyorum

☐ Sonucun <u>hastaya tıbbi, psikolojik ve sosyal etkilerini</u> göz önünde bulundurarak bilgi veriyorum

☐ <u>Hiçbir zaman</u> hastalarıma sonuç hakkında bilgi vermiyorum → Neden? (*lütfen yazınız*)………………………..

14. **Test yapılan kişiye sonucun açıklanmasını/açıklanmamasını hangi durumlarda tercih ediyorsunuz?** (Gerektiğinde birden fazla seçenek işaretleyebilirsiniz)**:**

☐ Kişinin veya ailesinin göreceği tıbbi, sosyal ya da ekonomik bir yarar olmadığında açıklamıyorum

☐ Kişinin dışlanma ve damgalanma endişesi olduğunda açıklamıyorum

☐ Sadece tanıyı doğruladığında açıklıyorum

☐ Kişinin kendi genetik yapısıyla ilgili olduğu için her durumda açıklıyorum

☐ ya da (*lütfen yazınız*) ……………………….

III. GENETİK BİLGİNİN KULLANILIŞINA İLİŞKİN TERCİHLERİNİZ

15. **Sizce kişinin genetik bilgileri diğer tıbbi bilgilerinden farklı mıdır?** ☐ Evet ☐ Hayır

16. **Sizce genetik test sonucunda elde edilen bilgi kime aittir?** (Lütfen tek seçenek işaretleyiniz)

☐ Bireye ait bir bilgidir ☐ Aileye ait bir bilgidir ☐ Etnik gruba ait bir bilgidir

☐ Devlete ait bir bilgidir ☐ İnsanlığa ait bir bilgidir

17. **Sizce bir yetişkine ait genetik test sonuçları o kişiden başka kimlerle paylaşılabilir?** (Gerektiğinde birden fazla seçenek işaretleyebilirsiniz)**:**

☐ Eşiyle / Partneriyle ☐ Genetik bağı olan aile üyeleriyle

☐ Sosyal bağı olan diğer kişilerle ☐ Sigorta şirketiyle

☐ İşvereniyle ☐ Diğer klinisyen meslektaşlarımla ☐ Hiç kimseyle

18. **Sizce reşit olmayan kişilere ait genetik bilgiler <u>kaç yaşından</u> itibaren kendileriyle paylaşılabilir? ....**

19. **Sizce kişi başkalarını da etkileyebilecek test sonucunu kimseyle paylaşmak istemediğinde, bu isteğine saygı gösterilmeli midir?**

☐ Evet ☐ Hayır

20. **Sizce hangi durum/durumlarda genetik bilgiden etkilenebilecek aile üyelerine açıklama yapılmalıdır?** (Gerektiğinde birden fazla seçenek işaretleyebilirsiniz)**:**

☐ Erken tanıyla hastalığın kontrol altına alınabileceği durumlarda
☐ Cinsel kimlik değişiminin söz konusu olduğu durumlarda
☐ Erken tedaviyle yaşam kalitesinin ya da sağkalım süresinin artırılabileceği durumlarda
☐ Kariyer tercihlerinin etkilenebileceği durumlarda
☐ Koruyucu önlemlerle yaşam kalitesinin ya da sağkalım süresinin artırılabileceği durumlarda
☐ Eş seçiminin etkilenebileceği durumlarda
☐ Üremeye ilişkin tercihlerin etkilenebileceği durumlarda
☐ Hiçbir etkisi olmasa da, aile üyelerinin de genetik yapısına ait bir bilgi olduğu için her durumda

21. **Sizce genetik test sonuçlarının hastaya kim tarafından açıklanması en uygundur?**

☐ Testi öneren klinisyen ☐ Testi yapan genetik uzmanı
☐ Özel eğitim almış genetik danışman ☐ ya da (*lütfen yazınız*) ……………….

22. **Sizce genetik testlerden edinilen bilgilerin gizliliği en iyi nasıl sağlanır?**

☐ Tüm tıbbi kayıtlar nasıl korunuyorsa genetik bilgiler de öyle korunmalıdır
☐ Test yapan özel kuruluş ya da devlet kuruluşu genetik bilgilerin korunması için özel önlemler almalıdır
☐ Devlet genetik bilgilerin korunmasını düzenlemeli ve denetlemelidir
☐ ya da (*lütfen yazınız*) ………………………………………………………………………..

23. **Genetik bilgilerin açıklanması ve paylaşılmasına ilişkin kurumsal ya da ulusal kurallara ihtiyaç duyuyor musunuz?**

☐ Evet ☐ Hayır

24. **Meslektaşlarınızla genetik bilgilerin açıklanması ve paylaşılmasının sosyal ve etik yönlerini tartışmaya ihtiyaç duyuyor musunuz?**

☐ Evet ☐ Hayır

25. **Sizce devlet genetik test yaptıracak herkese genetik danışmanlık sağlamalı mıdır?**

☐ Evet ☐ Hayır

IV. OLGULAR: Aşağıdaki olgular etik konuları göz önünde bulundurularak senaryolaştırılmış gerçek olgulardır. Hasta ve hekimlerin gerçek isimleri kullanılmamıştır. Lütfen kendinizi her bir vakadaki hekimin yerine koyarak size en uygun gelen ifadeyi işaretleyiniz.

OLGU A. *26 yaşındaki Nurgül prematüre bir bebek dünyaya getirir. Bebekte hipertelorizm, simian çizgisi, frau yarığı, endokardiyal yastık defekti, kronik akciğer hastalığı ve pulmoner hipertansiyon bulunmaktadır. Down sendromundan şüphelenen yenidoğan uzmanının önerdiği testle kromozom anomalisi belirlenir. Tekrar çocuk doğurmayı düşünen anne, ısrarla Down sendromunun nedeninin araştırılmasını, kendisine ve eşine test yapılmasını ister. Babanın gönülsüzlüğünü gören yenidoğan uzmanı, gelecekteki gebelikte yaptırabilecekleri prenatal tanı testleri hakkında bilgi verir. İki hafta sonra Nurgül ile onun aşırı ısrarına dayanamayan eşi Mahmut test için başvururlar. Test sonucunda babanın dengeli translokasyon taşıyıcısı olduğu belirlenir. Mahmut'un kendisinden daha genç ve çocuk sahibi olma potansiyeli olan birkaç kardeşi daha vardır. Dr. Gül dengeli taşıyıcılık bilgisini birinci derece yakınlarıyla ve tekrar çocuk doğurmayı planlayan eşiyle paylaşmasını önerir. Mahmut ise akrabalarıyla ilişkilerinin çok da iyi olmadığını, ayrıca tekrar çocuk sahibi olmayı da planlamadığını söyler. Bu nedenle diğer aile üyelerine ve eşine test sonucunun söylenmemesinde ısrar eder.*

26. **Hekim, Mahmut'un kararına saygı duymalıdır.**

☐ Katılıyorum ☐ Kararsızım ☐ Katılmıyorum

27. **Hekim, sonucu Mahmut'un eşine bildirmelidir.**

☐ Katılıyorum ☐ Kararsızım ☐ Katılmıyorum

28. **Hekim aile birliğini korumak adına Nurgül'e gerçeği açıklamamalıdır.**

☐ Katılıyorum ☐ Kararsızım ☐ Katılmıyorum

29. **Hekim, Mahmut'un kardeşlerini sonuçtan haberdar etmelidir.**

☐ Katılıyorum ☐ Kararsızım ☐ Katılmıyorum

30. **Mahmut'un, kardeşlerine açıklama yapma ve onları test yaptırmaya yönlendirme yükümlülüğü olmalıdır.**

☐ Katılıyorum ☐ Kararsızım ☐ Katılmıyorum

31. **Bu tür durumlarda devlet, ücretsiz test yapmakla yükümlü olmalıdır.**

☐ Katılıyorum ☐ Kararsızım ☐ Katılmıyorum

OLGU B. *21 yaşındaki Fevziye fiziksel görünümünde, cinsel organlarında ve duygularındaki farklılıklardan dolayı bir kadın doğum uzmanına başvurur. Psödohermafrodit olduğu belirlenen hastanın karyotip analizi sonucunda 46 XY ve 5-alfa reduktaz gen mutasyonuna sahip olduğu saptanır. Fevziye'yle sonuçlar hakkında görüşüldüğünde böyle bir sonuç beklediğini, zaten duygusal olarak da erkek gibi hissettiğini söyler. Dr. Erol, Fevziye'nin 16 yaşındaki kız kardeşi Şükrüye'de de fiziksel görünümünden dolayı benzer mutasyondan şüphelenir ve aynı testlerin ona da yapılmasını önerir. Çünkü mutasyonun 1:8 oranında diğer kardeşte de görülme ihtimali vardır. Fakat Fevziye bu öneriye şiddetle karşı çıkar. Kardeşi liseyi bitirdiğinde ona testleri yaptıracağına söz verir. Ancak Dr. Erol, test yapılmasının ertelenmesinin kardeşine zarar verebileceğini, kardeşinin duygusal sorunlar yaşayabileceğini açıklayarak test yapılması hakkında ısrar eder.*

32. Hekim, Fevziye'nin kararına saygı duymalıdır.

❍ Katılıyorum ❍ Kararsızım ❍ Katılmıyorum

33. Hekim dosya bilgilerinden aileye ulaşarak Şükrüye'ye de test yaptırılması gerektiğini söylemelidir.

❍ Katılıyorum ❍ Kararsızım ❍ Katılmıyorum

34. Hekim, Şükrüye'ye ulaşarak kendisine de abisine/ablasına yapılan testlerin yapılması gerektiğini söylemelidir.

❍ Katılıyorum ❍ Kararsızım ❍ Katılmıyorum

35. Fevziye kabul etmese dahi aile üyelerini uyarmak, hekimin yasal yükümlülüğü olmalıdır.

❍ Katılıyorum ❍ Kararsızım ❍ Katılmıyorum

36. Fevziye'nin, kardeşine açıklama yapma ve onu test yaptırmaya cesaretlendirme ödevi olmalıdır.

❍ Katılıyorum ❍ Kararsızım ❍ Katılmıyorum

37. Bu tür durumlarda devlet, ücretsiz test yapmakla yükümlü olmalıdır.

❍ Katılıyorum ❍ Kararsızım ❍ Katılmıyorum

OLGU C. *On beş aylık olan bebek Can dördüncü ayından beri solukluk, huzursuzluk, uykusuzluk, iyi kilo alamama yakınmalarıyla getirilmiştir. Öyküsünden birkaç kez kan nakli yapıldığı öğrenilir. Çocuk hematoloğu Dr. Nazlı tarafından talasemi majör tanısı konur. Taşıyıcı olan ebeveynler çocuklarının bu durumuna çok üzülmekle birlikte özellikle anne, ikinci bir çocuk doğurmayı planlamaktadır. Bu nedenle taşıyıcı olabilecek diğer aile üyelerinin bu konudan haberdar edilmesini istememektedir.*

38. Hekim, planladığı gebelik için anneyi prenatal tanı testi seçenekleri hakkında bilgilendirmelidir.

❍ Katılıyorum ❍ Kararsızım ❍ Katılmıyorum

39. Hekim, talasemi majör hastası bir başka çocuk dünyaya getirmemeye çifti ikna etmeye çalışmalıdır.

❍ Katılıyorum ❍ Kararsızım ❍ Katılmıyorum

40. Hekim, akrabalarını tanıyla ilgili bilgilendirmeleri hakkında bebek Can'ın ebeveynini uyarmalıdır.

❍ Katılıyorum ❍ Kararsızım ❍ Katılmıyorum

41. Çiftin akrabalarını uyardıklarından hekim emin olmalıdır.

❍ Katılıyorum ❍ Kararsızım ❍ Katılmıyorum

42. Çiftin akrabalarını uyarmak hekimin yasal ödevi olmalıdır.

❍ Katılıyorum ❍ Kararsızım ❍ Katılmıyorum

43. Devletin gelecek kuşakların sağlığını korumak adına bu testleri ücretsiz yaptırma yükümlülüğü olmalıdır.

❍ Katılıyorum ❍ Kararsızım ❍ Katılmıyorum

OLGU D. *14 yaşındaki Mustafa bir üniversite hastanesine tansiyon artışı nedeniyle başvurur. Mustafa yaşına göre kısa boyludur ve androjeni yüksektir. Fenotipi erkek olan çocuğun kromozom analizinden de 46XY olduğu belirlenir. Çocuk endokrinoloğu Dr. Figen, hastanın dosyasından onun bebekliğinde bir başka üniversite hastanesinde konjenital adrenal hiperplazi tanısı almış olduğunu öğrenir. Tanıyı koyan hastanedeki meslektaşıyla yaptığı görüşmede Mustafa'nın amcasının kızı olan Ayşe'nin de aynı tanıyı aldığını, fakat kız olarak yetiştirildiğini öğrenir. Bu bilgiyi Mustafa'nın ailesiyle paylaşır. Baba yıllardır konuşmadığı erkek kardeşiyle görüşerek Mustafa ile Ayşe'nin hastalığından söz eder. Bunun üzerine Ayşe'nin ailesi kendilerine ait bilgileri izinleri olmaksızın paylaştığı için tanıyı koyan üniversite hastanesine karşı dava açar.*

44. İlk tanıyı koyan hekim, bilgi sahiplerinin iznini almadan bu bilgileri paylaşmamalıydı.

❍ Katılıyorum ❍ Kararsızım ❍ Katılmıyorum

45. Dr. Figen, meslektaşından öğrendiği bilgileri hastasının ailesine açıklamamalıydı.

❍ Katılıyorum ❍ Kararsızım ❍ Katılmıyorum

46. Hekim, Ayşe'nin genetik bilgilerini sadece Mustafa'ya açıklamalıydı.

❍ Katılıyorum ❍ Kararsızım ❍ Katılmıyorum

47. Ayşe'nin ebeveyni, Mustafa'yı da ilgilendiren bu bilgileri daha önce kardeşinin ailesiyle paylaşmalıydı.

❍ Katılıyorum ❍ Kararsızım ❍ Katılmıyorum

48. Genetik bilgiler, meslektaşlar arasında olsa bile yasal düzenlemelere göre paylaşılmalıdır.

❍ Katılıyorum ❍ Kararsızım ❍ Katılmıyorum

49. Hastanenin/hekimlerin genetik bilgilerin emniyetini sağlama yükümlülüğü olmalıdır.

❍ Katılıyorum ❍ Kararsızım ❍ Katılmıyorum

İletmek istediğiniz bir olgu, bilgi veya not varsa lütfen yazınız. Sorularınız için aşağıdaki adreslerden bize ulaşabilirsiniz.

....

....

....

....

....

....

....

....

....

....

....

....

....

....

....

....

....

....

....

....

☺ Katkılarınız için çok te şekkür ederiz ☺

Arş. Gör. Aslıhan AKPINAR
Adres: Kocaeli Üniversitesi Tıp Fakültesi Tıp Tarihi ve Etik Anabilim Dalı, 41380 Umuttepe-İzmit
Telefon: 0-262-303-74-50
E-posta: aslyakcay@yahoo.com
