# Supplementary material for: Attitudes of physicians and patients towards disclosure of genetic information to spouse and first-degree relatives: a case study from Turkey
Source: BMC Med Ethics. 2014 May 16;15:39. doi: 10.1186/1472-6939-15-39 (PMC4029893; doi:10.1186/1472-6939-15-39)
Supplement: Additional file 2 — Physician’s Form in English. [file 1472-6939-15-39-S2.doc]

**ANNEX 1. PHYSICIAN’S QUESTIONNAIRE**

Dear Participant,

As from 1990’s, remarkable developments have been experienced in human genetics field. Such developments in clinic genetics, particularly in genetics on the other hand, lead to emergence of ethical problems with regards to particular issues as autonomy, privacy and confidentiality. Considerable ethical debates have started about by whom and under which circumstances can genetic information of an individual is procured. With this study we aimed to learn about the attitudes and preferences of physicians and patients about disclosure of and sharing personal genetic information. We aim to start an ethical debate about disclosure of personal genetic information and protection of confidentiality in national and international fields in line with the views of the parties and to be able to make some suggestions within this context in order for establishment of distinctive rules for Turkey.

***Thank you for your contributions.***

**I. PERSONAL INFORMATION**

1. Sex: Male Female

2. Age: ………………

3. Child: Have Don’t have

4. Area of specialization and if you have, minor area of specialization

5. How long have you been working as a specialist?

6. Type of the institution you work for (more than one option can be chosen):

State Hospital University/Training Hospital Private Hospital Clinic

7. How much information do you have about genetic researches?

Sufficient (good enough) Medium Level Insufficient

8. How important is religion in your life?

Important Neither important nor unimportant Unimportant

9. How can you describe your political view with one word? …………….

**II. YOUR PRACTICES RELATED TO GENETIC TESTING**

10. Do you recommend any tests to your patients during your daily practices?

Yes No (If your answer is No, please skip to Question no 15)

11. How often do you recommend genetic testing?

At least once a week At least once a month At least once every six months

Other (please indicate)

12. What are the sources for information related to genetic tests you recommend?

Medical Education Medical Specialty Education Scientific Journals I follow

Courses / Seminars Other (please indicate)

13. In which situations, do you inform your patients about the results of genetic tests

I always inform my patients about the results

I give information considering the medical, psychological and social impacts of the results on the patient

I never inform my patients about the results, Why? (please indicate)

14. In which situations, do you prefer disclosure/nondisclosure of the test results to patients? (If necessary, you may check more than one box)

I don’t disclose if there is no medical, social or economic benefit for the person or his/her family

I don’t disclose when it’s concerned about the exclusion or stigmatization of the person

I disclose only after diagnosis is verified

I disclose in every situation since it’s about the person’s own genetic constitution

Other (please indicate)

**III. YOUR PREFERENCES REGARDING USAGE OF GENETIC INFORMATION**

15. Do you think a person’s genetic information is different from other medical information?

Yes No

16. To whom do you think information obtained by genetic test belong?

To the person To the family To the ethnic group

To state To humanity

17. With whom do you think genetic test results of an adult can be shared other than such adult? (If necessary, you may check more than one box)

His/her spouse/partner Family members who have genetic bond with the person

Other persons who have social bonds with the person Insurance company

Employer Other clinician colleagues of me No-one

18. As from what age do you think genetic information of a minor can be shared with him/her? ………………..

19. When a person doesn’t want to share results of a test that might affect others, do you think this request should be respected?

Yes No

20. In which situation(s) do you think it’s acceptable to inform other family members who might be affected by this information? (If needed, you may choose more than one option)

In situations in which disorder can be taken under control by early diagnosis

In situations in which quality or time of life can be increased by early treatment

In situations in which quality or time of life can be increased by protective measures

In situations in which gender reassignment is in question

In situations in which career choices might be effected

In situations in which spouse choices might be effected

In situations in which choices regarding reproduction might be effected

In every situation in which information regarding my family members is found even if there is no effect

21. By whom should the test results be announced to the patient?

The doctor who suggested the test

The specialist who will carry out the test

Genetic advisor who has a special training

Other (please indicate) ………………………

22. What is the best way to maintain confidentiality of genetic test information?

Genetic information should be protected/preserved as all other medical records are protected

Testing private institution or public body should take special precautions to protect genetic information

Government should regulate and audit protection of genetic information

23. Do you need institutional or national policies about disclosure and sharing genetic information?

Yes No

24. Do you need to discuss social and ethical aspects of genetic testing?

Yes No

25. Do you think government should provide genetic counseling to anyone who will make genetic test?

Yes No

**IV. CASES:** Below mentioned cases are the cases scripted considering the ethic subjects. Real patient or doctor names were not used. Please try to put yourself in the person’s place who has taken the test and choose the most appropriate option.

CASE A: 26-year-old Nurgül gave birth to a premature baby. The baby had hypertelorism, simian crease, endocardial cushion defects, chronic lung disease and pulmonary hypertension. A chromosome anomaly was detected in the test which had been suggested by Dr Elif, the neonatalogist, who suspected that the baby might have Down syndrome. The mother, who was hoping to have another baby in the future, insisted that the cause of the Down syndrome be investigated and asked for tests to be carried out both on her and on her husband. Realizing that the father was unwilling to participate, the neonatalogist Dr. Elif gave information to the spouses about prenatal diagnostic tests which could be carried out in the course of a future pregnancy. Two weeks later, Nurgül and her husband Mahmut, who could not withstand his wife’s determination, applied to have the test. Mahmut was identified by the test results as a balanced translocation carrier. Mahmut has younger siblings, all of whom may wish to have children in the future. Dr. Elif suggested to Mahmut that it would be helpful to them if he were to share this information about his condition with his first degree relatives and his wife who was keen to start a second pregnancy. However, Mahmut said that his communication with his relatives was not at all good anyway, and he was not planning to have a second baby. For these reasons, he refused to tell his family members, or his wife, about the test result.

26. Doctor should respect father’s decision

I agree I neither agree nor disagree (neutral) I disagree

27 Doctor should inform Nurgül even if the father doesn’t want

I agree I neither agree nor disagree (neutral) I disagree

28. Doctor should not disclose the truth in order to protect the unity of family

I agree I neither agree nor disagree (neutral) I disagree

29. Doctor should also inform the father’s brothers

I agree I neither agree nor disagree (neutral) I disagree

30. Father should be obliged to inform his brothers and direct them to be tested too

I agree I neither agree nor disagree (neutral) I disagree

31. In such situations, state must be obliged to make the tests free of charge

I agree I neither agree nor disagree (neutral) I disagree

**CASE B.** Fevziye, 21 years old, consulted a gynecologist due to differences in her genitals and in feelings. It was found that the patient was and pseudohermaphrodite and in karyotyping, it was determined that the patient had 46 XY and 5-alfa reductase gene mutation. When the results were discussed with Fevziye, she mentioned that she has expected such a result and she already emotionally felt that she was a man. Dr Erol suspected that Sükrüye, 16 years old sister of Fevziye might have same mutation due to her appearance and suggested the same test to be applied for her. Because there was the 1/8 possibility that her sister might also have the mutation. Fevziye strongly objected this suggestion. She promised that she would make the tests applied after her sister graduated from high-school. However, Dr. Erol insisted that the tests should be applied because a delay in the tests might hurt her sister and she might have emotional problems.

32. The doctor should respect the decision of Fevziye

I agree I neither agree nor disagree (neutral) I disagree

33. The doctor should contact the family members and tell them that tests should be applied to Sükrüye

I agree I neither agree nor disagree (neutral) I disagree

34. The doctor should contact Sükrüye and tell her that same tests applied to her sister/brother should be applied to her too

I agree I neither agree nor disagree (neutral) I disagree

35. It should be the doctor’s legal obligation to inform the family even if Fevziye doesn’t accept

I agree I neither agree nor disagree (neutral) I disagree

35. It should be Fevziye’s responsibility to inform her sister and to encourage her about the test

I agree I neither agree nor disagree (neutral) I disagree

36. In such situations, state must be obliged to make the tests free of charge

I agree I neither agree nor disagree (neutral) I disagree

**CASE C.** Can, a fifteen months infant, was brought to the hospital with the complaints of breathlessness, restlessness, sleeplessness since he was four months. It was learnt that blood transfusion was made a couple of times. Child’s hematologist Dr. Nazli made thalassemia major diagnosis. As disorder carrying parents feel very sad about such situation of their children, particularly the mother was planning to have a second baby. Therefore, she requested the other members of the family who might be carriers not to be informed about that issue.

37. The doctor should give information about prenatal diagnosis test to the mother for the pregnancy she was planning

I agree I neither agree nor disagree (neutral) I disagree

38. The doctor should try to convince the couple that they should not have a second baby which will also have thalassemia major disorder.

I agree I neither agree nor disagree (neutral) I disagree

39. The doctor should warn the couple about informing their relatives about the disorder

I agree I neither agree nor disagree (neutral) I disagree

40. The doctor should make sure that the relatives of the couple will be warned

I agree I neither agree nor disagree (neutral) I disagree

41. It should be the doctor’s legal obligation to inform the relatives of the couple

I agree I neither agree nor disagree (neutral) I disagree

42. State should be obliged to make such tests free of charge in order to protect the health of posterity

I agree I neither agree nor disagree (neutral) I disagree

**CASE D.** Mustafa, 14 years old, consulted to a university hospital due to increasing blood pressure. Mustafa was short in terms of his age and had high androgynous. His phenotype was male and through the chromosome analysis, it was determined that he was 46XY. Child endocrinologist Dr. Figen learnt from the patient’s file that he had congenital adrenal hyperplasia diagnosis from a different university hospital which made test during his infancy. Upon meeting with his colleague who had made the diagnosis, the doctor learnt that Mustafa’s cousin Ayse had the same diagnosis as well but raised as female. The doctor shared this information with Mustafa. Mustafa’s father saw his brother with him who hasn’t been talking for years and talked about the disorder Mustafa and Ayse had. Thereupon, parents of Ayse entered a lawsuit against the university hospital which made the diagnosis on the grounds that the hospital has shared information belonged to the parents without their permission/consent.

44. Doctor who has made the first diagnosis shouldn’t have shared the information without prior permission of related persons

I agree I neither agree nor disagree (neutral) I disagree

45. Dr. Figen shouldn’t have presented the information she has received from her colleague to the patient’s family

I agree I neither agree nor disagree (neutral) I disagree

46. The doctor should have told genetic information of Ayse only to Mustafa

I agree I neither agree nor disagree (neutral) I disagree

47. Parent of Ayse should have shared the information which also concerns Mustafa with the family of his brother

I agree I neither agree nor disagree (neutral) I disagree

48. Genetic information should be shared in accordance with legal regulations, even when it’s between colleagues

I agree I neither agree nor disagree (neutral) I disagree

49. Hospitals/Doctors should be obliged to secure the genetic information of patients

I agree I neither agree nor disagree (neutral) I disagree

Please write down any case, information or remark you would like to state. You can contact us at below mentioned addresses:

Thank you very much for your contributions.

Res. Asst. Aslihan AKPINAR

Address: Kocaeli University, Medical Faculty, Department of Medical History and Ethics, 41380 Umuttepe – Izmit

Tel: 0 262 303 74 50

E-mail: [aslyakcay@yahoo.com](mailto:aslyakcay@yahoo.com)
