# Supplementary material for: Attitudes of physicians and patients towards disclosure of genetic information to spouse and first-degree relatives: a case study from Turkey
Source: BMC Med Ethics. 2014 May 16;15:39. doi: 10.1186/1472-6939-15-39 (PMC4029893; doi:10.1186/1472-6939-15-39)
Supplement: Additional file 3 — Patient’s Form in Turkish. [file 1472-6939-15-39-S3.pdf]

Genetik Bilginin Kullanılmasında Etik: Tarafların Tutum ve Tercihleri - *Hasta Formu*

Değerli Katılımcı,

Son 20 yılda insan genetiği alanında olağanüstü gelişmeler yaşanmaktadır. Özellikle genetik hastalıkların tanımlanmasında yaşanan bu gelişmeler bazı etik/ahlak sorunlarının ortaya çıkmasına neden olmaktadır. Elde edilen genetik bilginin kime ait olduğu ve kimlerle paylaşılması gerektiği tartışmaların merkezindedir. Planladığımız bu çalışmayla, testi öneren hekimler ile test yaptıran kişilerin genetik bilgilerin açıklanması ve paylaşılması hakkındaki tutum ve tercihlerini öğrenmek amaçlanmıştır. Çalışmayla elde edilecek bilgiler doğrultusunda ülkemize özgü kuralların oluşumuna katkı sunabilmeyi hedeflemekteyiz.

Katkılarınız için teşekkür ederiz.

Prof. Dr. Nermin ERSOY (Tez Danışmanı), Arş. Gör. Aslıhan AKPINAR (Tez Yürütücüsü)

I. DEMOGRAFİK ÖZELLİKLER

1. **Cinsiyetiniz :** ☐ Erkek ☐ Kadın *2.* **Yaşınız: ....**

3. **Medeni durumunuz:** ☐ Evli ☐ Bekar *4.* **Çocuğunuz:** ☐ Var ☐ Yok

5. **Dinin yaşamınızdaki yeri?** ☐ Önemlidir ☐ Ne önemli ne önemsizdir ☐ Önemli değildir

6. **Politik görüşünüzü tek kelimeyle nasıl ifade edersiniz? ………………**

7. **Herhangi bir işte çalışıyor musunuz?** ☐ Evet ☐ Hayır *8.* **Mesleğiniz? ……………………….**

9. **Eğitim durumunuz?** ☐ Okur-yazar değil ☐ Okur-yazar ☐ İlkokul ☐ Ortaokul
☐ Lise ☐ Ön-Lisans ☐ Lisans ☐ Diğer …………………..

10. **Genetik hastalıklar ve genetik testler konusunda ne kadar bilgi sahibisiniz?**
☐ Yeterli ☐ Orta düzeyde ☐ Yetersiz ☐ Hic bilgim yok

II. TEST BİLGİSİ

11. **Size yaptıracağınız test hakkında bilgi verildi mi?**
☐ Evet ☐ Hayır

12. **Evet ise, kim tarafından verildi?**
☐ Testi öneren doktor ☐ Testi yapacak olan uzman ☐ ya da (*lütfen yazınız*) …………………

13. **Bu genetik testin sonucunun size açıklanmasını ister misiniz?**
☐ Evet ☐ Hayır *13a.* **Neden?..**

III. GENETİK BİLGİNİN KULLANILMASI İLİŞKİN TERCİHLER

14. **Sizce genetik bilgiler hastalık ve tedavilerle ilgili tıbbi bilgilerden farklı mıdır?**
☐ Evet ☐ Hayır *14a.* **Neden?..**

15. **Sizce genetik test sonucunda elde edilen bilgi kime aittir?**
☐ Bireye ait bir bilgidir. ☐ Aileye ait bir bilgidir. ☐ Etnik gruba ait bir bilgidir.
☐ Devlete ait bir bilgidir. ☐ İnsanlığa ait bir bilgidir.

16. **Yaptırdığınız genetik testin sonuçlarının kimlerle paylaşılmasına izin verirsiniz?** (Gerektiğinde birden fazla seçenek işaretleyebilirsiniz)**:**
☐ Eşimle / Partnerimle ☐ Genetik bağım olan aile üyelerimle (bir sınırlandırmanız var mı?..................
☐ Sosyal bağım olan diğer kişilerle ☐ Sigorta şirketimle ....
☐ İşverenimle ☐ Diğer doktorlarla ....
☐ Hiçbiriyle

17. **Sizce reşit olmayan kişilere ait genetik bilgiler kaç yaşından itibaren kendileriyle paylaşılabilir? ....**

18. **Test sonucunda ailede başkalarını da ilgilendirebilecek bir bir bilgi elde edilse bile, onlarla paylaşmama hakkınız olduğuna inanıyor musunuz?**
☐ Evet ☐ Hayır

19. **Bu bilgiden etkilenebilecek olan aile üyelerinize hangi durum/durumlarda açıklama yapılmasını kabul edersiniz?** (Gerektiğinde birden fazla seçenek işaretlenebilir)**:**
☐ Erken tanı ile hastalığın kontrol altına alınabileceği durumlarda
☐ Erken tedavi ile yaşam kalitesinin ya da süresinin arttırılabileceği durumlarda
☐ Koruyucu önlemlerle yaşam kalitesinin ya da süresinin artırılabileceği durumlarda
☐ Cinsel kimlik değişiminin sözkonusu olduğu durumlarda
☐ Kariyer tercihlerinin etkilenebileceği durumlarda
☐ Eş seçiminin etkilenebileceği durumlarda
☐ Üremeye ilişkin tercihlerin etkilenebileceği durumlarda
☐ Hiçbir etkisi olmasa da aile üyelerimin de genetik yapısına ait bir bilgi olduğu için her durumda

20. Genetik test sonuçları hastaya kim tarafından açıklanmalıdır?

- Testi öneren doktor
- Testi yapan uzman
- Özel eğitim almış genetik danışman **ya da** ……………………

21. Genetik test bilgilerinin gizliliği en iyi nasıl sağlanır?

- Tüm tıbbi kayıtlar nasıl korunuyorsa genetik bilgiler de öyle korunmalıdır
- Test yapan özel kuruluş ya da devlet kuruluşu genetik bilgilerin korunması için özel önlemler almalıdır
- Devlet genetik bilgilerin korunmasını düzenlemeli ve denetlemelidir

22. Genetik bilgilerin nasıl kullanılacağı hakkında o sağlık kurumunun ya da devletin kuralları olmalı mıdır?

- Evet
- Hayır

23. Genetik danışmanlığın ne olduğunu biliyor musunuz? Evet / Hayır

23. Devlet genetik test yaptıracak olan herkese genetik danışmanlık sağlamalı mıdır?

- Evet
- Hayır

IV. OLGULAR: Aşağıdaki olgular etik konuları göz önünde bulundurularak senaryolaştırılmış olgulardır. Gerçek hasta veya hekim isimleri kullanılmamıştır. Lütfen kendinizi her bir vakada test yaptıran kişinin yerine koyarak size uygun gelen ifadeyi seçiniz.

OLGU A. *26 yaşındaki Nurgül'ün bebeği erken doğmuştur. Bebeğin Down sendromu olmasından şüphelenen doktor test önerir ve test sonucunda doktorun şüphesi doğrulanır. Down sendromunun nedenleri arasında annenin gebelik yasının yuksek olması, anne veya babanin genetik olarak taşıdığı bazı bozukluklar da sayılabilir. İkinci bir çocuk sahibi olmak isteyen Nurgül ısrarla bebeklerinin neden Down sendromu olduğunun araştırılmasını, kendisine ve eşine genetik testler yapılmasını ister. Baba gönülsüzce de olsa eşinin aşırı ısrarına dayanamayarak test yapılmasını kabul eder. Test sonucunda babanın genetik bozukluğunun bebekte hastalığın olmasına neden olduğu öğrenilir. 30 yaşındaki babanın kendisinden daha genç kardeşleri vardır. Doktor babaya eşine ve kardeşlerine durumu hakkında bilgi vermesini önerir. Fakat koca başka çocuk istemediğini, kardeşleriyle de arasının iyi olmadığını söyleyerek bilgi vermeyi reddeder.*

24. Doktor, babanın kararına saygı duymalıdır.

- Katılıyorum
- Kararsızım
- Katılmıyorum

25. Doktor, baba istemese bile sonucu Nurgül'e bildirmelidir.

- Katılıyorum
- Kararsızım
- Katılmıyorum

26. Doktor aile birliğini korumak adına Nurgül'e gerçeği açıklamamalıdır.

- Katılıyorum
- Kararsızım
- Katılmıyorum

27. Doktor, babanın kardeşlerini de sonuçtan haberdar etmelidir.

- Katılıyorum
- Kararsızım
- Katılmıyorum

28. Babanın, kardeşlerine açıklama yapma ve onları test yaptırmaya yönlendirme yükümlülüğü olmalıdır.

- Katılıyorum
- Kararsızım
- Katılmıyorum

29. Bu tür durumlarda devlet, ücretsiz test yapmakla yükümlü olmalıdır.

- Katılıyorum
- Kararsızım
- Katılmıyorum

OLGU B. *21 yaşındaki Fevziye bedensel görünümünde, cinsel organlarında ve duygularındaki farklılıklardan dolayı bir kadın doğum uzmanına başvurur. Yalancı çift cinsiyetli olduğu belirlenen hastaya yapılan test sonucunda erkek kromozomlarına sahip olduğu görülür. Fakat erkek cinsel organının gelişmesine engel olan bir bozukluğu vardır. Fevziye ile sonuçlar hakkında görüşüldüğünde böyle bir sonuç beklediğini, zaten duygusal olarak da erkek gibi hissettiğini söyler. Doktor, Fevziye'nin 16 yaşındaki kız kardeşi Şükrüye'de de fiziksel görünümünden dolayı benzer durumdan şüphelenir ve aynı testlerin ona da yapılmasını önerir. Çünkü bozukluğun 1:8 oranında diğer kardeşte de görülme ihtimali vardır. Fakat Fevziye bu öneriye şiddetle karşı çıkar. Kardeşi liseyi bitirdiğinde ona testleri yaptıracağına söz verir. Ancak doktor, test yapılmasının ertelenmesinin kardeşine zarar verebileceğini, kardeşinin duygusal sorunlar yaşayabileceğini açıklayarak test yapılması için ısrar eder.*

30. Doktor, Fevziye'nin kararına saygı duymalıdır.

- Katılıyorum
- Kararsızım
- Katılmıyorum

31. Doktor dosya bilgilerinden aileye ulaşarak Şükrüye'ye de test yaptırılması gerektiğini söylemelidir.

- Katılıyorum
- Kararsızım
- Katılmıyorum

32. Doktor, Şükrüye'ye ulaşarak kendisine de abisine/ablasına yapılan testlerin yapılması gerektiğini söylemelidir.

- Katılıyorum
- Kararsızım
- Katılmıyorum

33. Doktorun, Fevziye kabul etmese dahi aile üyelerini uyarmakla ilgili yasal ödevi olmalıdır.

- Katılıyorum
- Kararsızım
- Katılmıyorum

34. Fevziye'nin, kardeşine açıklama yapma ve onu test yaptırmaya cesaretlendirme ödevi olmalıdır.

- Katılıyorum
- Kararsızım
- Katılmıyorum

35. Bu tür durumlarda devlet, testi ücretsiz yapmakla yükümlü olmalıdır.

- Katılıyorum
- Kararsızım
- Katılmıyorum

Sayfa 2/3 ➔

OLGU C. *On beş aylık olan Bebek Can dördüncü ayından beri solukluk, huzursuzluk, uykusuzluk, iyi kilo alamama yakınmalarıyla doktora getirilmiştir. Öyküsünden birkaç kez kan nakli yapıldığı öğrenilir. Çocuk doktoru tarafından ağır bir kan hastalığı olan talasemi majör tanısı konur. Bu kalıtımsal hastalık taşıyıcı olan bireylerde kendini göstermez. Ancak hem anneden hem babadan hastalık genlerini almış olan çocuklarda ortaya çıkar. Taşıyıcı olanların kan bağı olan akrabaları da taşıyıcı olabilir. Anne-baba çocuklarının bu durumuna çok üzülmekle birlikte ailenin olumsuz baskısını engellemek için onlara bilgi verilmesini istememektedirler.*

36. Doktor, ilerideki gebeliğinde yaptırabileceği testler hakkında anneyi bilgilendirmelidir.

🕯 Katılıyorum 🕯 Kararsızım 🕯 Katılmıyorum

37. Doktor, çifti talasemi majör hastası bir başka çocuk dünyaya getirmemeye ikna etmeye çalışmalıdır.

🕯 Katılıyorum 🕯 Kararsızım 🕯 Katılmıyorum

38. Doktor, anne-babayı, akrabalarını hastalık hakkında bilgilendirmeleri için uyarmalıdır.

🕯 Katılıyorum 🕯 Kararsızım 🕯 Katılmıyorum

39. Doktor, çiftin akrabalarını uyardığından emin olmalıdır.

🕯 Katılıyorum 🕯 Kararsızım 🕯 Katılmıyorum

40. Doktorun çiftin akrabalarını uyarmakla ilgili yasal ödevi olmalıdır.

🕯 Katılıyorum 🕯 Kararsızım 🕯 Katılmıyorum

41. Devlet çocukların sağlığını korumak adına bu testleri ücretsiz yaptırmalıdır.

🕯 Katılıyorum 🕯 Kararsızım 🕯 Katılmıyorum

OLGU D. *14 yaşındaki Mustafa bir üniversite hastanesine tansiyon artışı ile başvurur. Mustafa yaşına göre kısa boyludur, gelişme geriliği olduğunu düşünen doktor kromozom analizi yaptırır. Test sonucunda Mustafa'nın erkek kromozomları olduğu belirlenir. Çocuk doktoru hastanın bebekliğinde bir başka üniversite hastanesinde doğumsal adrenal hiperplazi tanısı almış olduğunu öğrenir. Bu hastalık böbrek üstü bezlerinin aşırı büyümesi ve buradaki bazı enzimlerin eksikliği sonucu meydana gelir ve yalancı çift cinsiyetliliğe neden olur. Doktor ilk tanıyı koyan hastanedeki meslektaşıyla yaptığı görüşmede Mustafa'nın amcasının kızının da aynı tanıyı aldığını fakat kız olarak yetiştirildiğini öğrenir. Doktor bu bilgiyi Mustafa'nın ailesiyle paylaşır. Baba yıllardır konuşmadığı erkek kardeşiyle görüşerek çocuklarının benzer hastalığından söz eder. Bunun üzerine amca kendilerine ait bilgileri izinleri olmadan paylaştığı için ilk tanıyı koyan üniversite hastanesine karşı dava açar.*

42. İlk tanıyı koyan doktor bilgi sahiplerinin iznini almadan bu bilgileri paylaşmamalıydı.

🕯 Katılıyorum 🕯 Kararsızım 🕯 Katılmıyorum

43. Doktor meslektaşından öğrendiği bilgileri hastasının ailesine açıklamamalıydı.

🕯 Katılıyorum 🕯 Kararsızım 🕯 Katılmıyorum

44. Doktor, amca kızının genetik bilgilerini sadece Mustafa'ya açıklamalıydı.

🕯 Katılıyorum 🕯 Kararsızım 🕯 Katılmıyorum

45. Amca, Mustafa'yı da ilgilendiren bu bilgileri daha önce kardeşiyle paylaşmalıydı.

🕯 Katılıyorum 🕯 Kararsızım 🕯 Katılmıyorum

46. Genetik bilgiler, doktorlar arasında olsa bile yasal düzenlemelere göre paylaşılmalıdır.

🕯 Katılıyorum 🕯 Kararsızım 🕯 Katılmıyorum

47. Hastanenin/hekimlerin genetik bilgilerin emniyetini sağlama yükümlülüğü olmalıdır.

🕯 Katılıyorum 🕯 Kararsızım 🕯 Katılmıyorum

---

48. Hasta hangi uzmanlık dalı tarafından yönlendirilmiştir? 🕯 Kadın - doğum 🕯 Çocuk Hastalıkları

49. Yaptırılacak test türü nedir? 🕯 Moleküler genetik 🕯 Prenatal test 🕯 Sitogenetik

50. Hastanın …… 🕯 Kendisi 🕯 Annesi 🕯 Babası … … **ile görüşülmüştür.**

51. Şüphe edilen durum nedir? **52. Hastanın sigorta türü nedir?** 🕯 Özel 🕯 Devlet

---

İletmek istediğiniz bir bilgi veya not varsa lütfen yazınız.

Katkılarınız için çok teşekkür ederiz

…………………………………………………………………………………………………
…………………………………………………………………………………………………
…………………………………………………………………………………………………
…………………………………………………………………………………………………

☺ Katkılarınız için çok te şekkür ederiz ☺

Arş. Gör. Aslıhan AKPINAR
Adres: Kocaeli Üniversitesi Tıp Fakültesi Tıp Tarihi ve Etik Anabilim Dalı, 41380 Umuttepe-İzmit
Telefon: 0-262-303-74-50
E-posta: aslyakcay@yahoo.com
