# Supplementary material for: Attitudes of physicians and patients towards disclosure of genetic information to spouse and first-degree relatives: a case study from Turkey
Source: BMC Med Ethics. 2014 May 16;15:39. doi: 10.1186/1472-6939-15-39 (PMC4029893; doi:10.1186/1472-6939-15-39)
Supplement: Additional file 4 — Patient’s Form in English. [file 1472-6939-15-39-S4.doc]

**ANNEX 2. PATIENT’S FORM**

Dear Participant,

For the last 20 years, there have been remarkable developments in human genetics field. These developments, particularly ones experienced in diagnosis of genetic disorders, lead to some ethical/moral issues. The focus of the debates is on to whom the genetic information belongs and with whom it should be shared. With this study, it’s aimed to learn the attitudes and preferences of physicians and patients about disclosure and sharing of genetic information. In line with the information to be obtained in this study, we aim to make a contribution to formation of rules for Turkey.

**I. DEMOGRAPHIC INFORMATION**

1. Sex: Male Female

2. Age: ………………….

3. Marital Status: Married Single

4. Child: Have Don’t have

5. How important is religion in your life?

Important Neither important nor unimportant Unimportant

6. How can you describe your political view with one word? …………….

7. Are you employed? Yes No

8. Occupation: …………………………

9. Educational Background:

Illiterate Literate Elementary School Secondary School

High School Associate Degree Undergraduate Other …………….

10. How much information do you have about genetic disorders and genetic tests?

Sufficient (good enough) Medium Level Insufficient None at all

**II. TEST INFORMATION**

11. Were you informed about the test to be conducted?

Yes No

12. If yes, by whom?

The doctor who suggested the test

The specialist who will carry out the test

Other (please indicate) ………………………

13. Would like to learn the result of the genetic test to be made?

Yes No 13a. Why? ……………………………..

**III. PREFERENCES WITH REGARDS TO USAGE OF GENETIC INFORMATION**

14. Do you think genetic information is different from the other medical information regarding disorders and cures?

Yes No 14a. Why? ………………………

15. To whom do you think result to be obtained by the test belong?

To the person To the family To the ethnic group

To state To humanity

16. With whom would you permit your test results to be shared? (If needed, you may choose more than one option)

My spouse/My partner My family members with whom I have genetic bond (do you have a limitation)

Other people with whom I have social bonds

My insurance company My employer Other doctors

No one

17. As from what age do you think genetic information of a minor can be shared with him/her? …………………..

18. Do you believe that you have right not to share information to be obtained as a result of the test with your family members, even when the results concern them too?

Yes No

19. In which situation(s) do you think it’s acceptable to inform other family members who might be affected by this information? (If needed, you may choose more than one option)

In situations in which disorder can be taken under control by early diagnosis

In situations in which quality or time of life can be increased by early treatment

In situations in which quality or time of life can be increased by protective measures

In situations in which gender reassignment is in question

In situations in which career choices might be effected

In situations in which spouse choices might be effected

In situations in which choices regarding reproduction might be effected

In every situation in which information regarding my family members is found even if there is no effect

20. By whom should the test results be announced to the patient?

The doctor who suggested the test

The specialist who will carry out the test

Genetic advisor who has a special training

Other (please indicate) ………………………

21. What is the best way to maintain secrecy of genetic test information?

Genetic information should be protected/preserved as all other medical records are protected

Testing private institution or public body should take special precautions to protect genetic information

Government should regulate and audit protection of genetic information

22. Should there be any rules of related health institution or government regarding how genetic information will be used?

Yes No

23. Do you know what genetic counseling is?

Yes No

24. Do you think government should provide genetic counseling to anyone who will make genetic test?

Yes No

**IV. CASES:** Below mentioned cases are the cases scripted considering the ethic subjects. Real patient or doctor names were not used. Please try to put yourself in the person’s place who has taken the test and choose the most appropriate option.

**CASE A:** Nurgül, age 26, had a premature baby. Doctor, with the suspect that baby might have Down syndrome, suggested a test and the result of the test confirmed that Doctor’s suspect was right. Having high gestational age and some disorders father or mother might be carrying can be counted as two of the reasons of Down syndrome. Since Nurgül wanted to have a second baby, she insisted on searching for why the baby had Down syndrome and requested genetic tests to be applied to her and her spouse. Father reluctantly accepted the test upon insistence of his spouse. As a result of the test, it was found out that genetic disorder of the father caused baby’s disorder. Father, aged 30, had younger brothers. Doctor suggested the father to inform his spouse and brothers about the situation. But father refused to inform them claiming that he didn’t want any other baby and he didn’t get along well with his brothers.

25. Doctor should respect father’s decision

I agree I neither agree nor disagree (neutral) I disagree

26. Doctor should inform Nurgül even if the father doesn’t want

I agree I neither agree nor disagree (neutral) I disagree

27. Doctor should not disclose the truth in order to protect the unity of family

I agree I neither agree nor disagree (neutral) I disagree

28. Doctor should also inform the father’s brothers

I agree I neither agree nor disagree (neutral) I disagree

29. Father should be obliged to inform his brothers and direct them to be tested too

I agree I neither agree nor disagree (neutral) I disagree

30. In such situations, state must be obliged to make the tests free of charge

I agree I neither agree nor disagree (neutral) I disagree

CASE B. Fevziye, 21 years old, consulted a gynecologist due to differences in her physical appearance, genitals and in her feelings. It was determined that the patient had pseudohermaphrodism and seen upon the tests that she had male chromosomes. But she had a disorder which prevented growth of male genitals. When the results were discussed with Fevziye, she mentioned that she has expected such a result and she already emotionally felt that she was a man. The doctor suspected that Sükrüye, 16 years old sister of Fevziye might have same mutation due to her appearance and suggested the same test to be applied for her. Because there was the 1/8 possibility that her sister might also have the mutation. Fevziye strongly objected this suggestion. She promised that she would make the tests applied after her sister graduated from high-school. However, Dr. Erol insisted that the tests should be applied because a delay in the tests might hurt her sister and she might have emotional problems.

31. The doctor should respect the decision of Fevziye

I agree I neither agree nor disagree (neutral) I disagree

32. The doctor should contact the family members and tell them that tests should be applied to

Sükrüye

I agree I neither agree nor disagree (neutral) I disagree

33. The doctor should contact Sükrüye and tell her that same tests applied to her sister/brother should be applied to her too

I agree I neither agree nor disagree (neutral) I disagree

34. It should be the doctor’s legal obligation to inform the family even if Fevziye doesn’t accept

I agree I neither agree nor disagree (neutral) I disagree

35. It should be Fevziye’s responsibility to inform her sister and to encourage her about the test

I agree I neither agree nor disagree (neutral) I disagree

36. In such situations, state must be obliged to make the tests free of charge

I agree I neither agree nor disagree (neutral) I disagree

CASE C. Can, a fifteen months infant, was brought to the hospital with the complaints of breathlessness, restlessness, sleeplessness since he was four months. It was learnt that blood transfusion was made a couple of times. Child’s doctor made thalassemia major diagnosis, a heavy blood disorder. This disorder doesn’t show itself at carrying individuals. But it occurs at the children who have received the disorderd genes both from mother and from father. Relatives of carrying individuals might also be carrying persons. Even though parents felt very sad about their child, they didn’t want to inform the other family members in order to prevent negative pressure to be imposed by them.

37. The doctor should give information about prenatal diagnosis test to the mother for the pregnancy she was planning

I agree I neither agree nor disagree (neutral) I disagree

38. The doctor should try to convince the couple that they should not have a second baby which will also have thalassemia major disorder.

I agree I neither agree nor disagree (neutral) I disagree

39. The doctor should warn the couple about informing their relatives about the disorder

I agree I neither agree nor disagree (neutral) I disagree

40. The doctor should make sure that the relatives of the couple will be warned

I agree I neither agree nor disagree (neutral) I disagree

41. It should be the doctor’s legal obligation to inform the relatives of the couple

I agree I neither agree nor disagree (neutral) I disagree

42. State should be obliged to make such tests free of charge in order to protect the health of posterity

I agree I neither agree nor disagree (neutral) I disagree

**CASE D.** Mustafa, 14 years old, consulted to a university hospital due to increasing blood pressure. Mustafa was short in terms of his age and the doctor made chromosome analysis since he thought there might be growth deficiency. As a result of the test, it was seen that Mustafa had male chromosomes. Child doctor learnt that the patient had congenital adrenal hyperplasia diagnosis from a different university hospital which made test during his infancy. This disorder occurs as a result of excessive growth of suprarenal glands and lack of some of enzymes there and causes pseudohermaphrodism. Upon meeting with his colleague who had made the diagnosis, the doctor learnt that Mustafa’s cousin had the same diagnosis as well but raised as female. The doctor shared this information with Mustafa’s parents. Mustafa’s father saw his brother with him who hasn’t been talking for years and talked about the disorder both their children had. Thereupon, parents of Ayse entered a lawsuit against the university hospital which made the diagnosis on the grounds that the hospital has shared information belonged to the parents without their permission/consent.

43. Doctor who has made the first diagnosis shouldn’t have shared the information without prior permission of related persons

I agree I neither agree nor disagree (neutral) I disagree

44. The doctor shouldn’t have presented the information she has received from her colleague to the patient’s family

I agree I neither agree nor disagree (neutral) I disagree

45. The doctor should have told genetic information of the patient only to Mustafa

I agree I neither agree nor disagree (neutral) I disagree

46. Uncle should have shared the information which also concerns Mustafa with the family of

his brother

I agree I neither agree nor disagree (neutral) I disagree

47. Genetic information should be shared in accordance with legal regulations, even when it’s between colleagues

I agree I neither agree nor disagree (neutral) I disagree

48. Hospitals/Doctors should be obliged to secure the genetic information of patients

I agree I neither agree nor disagree (neutral) I disagree

48. By which medical specialty the patient was directed to test?

Gynecology Pediatry

49. What is the type of the test to be made?

Molecular genetics Prenatal Testing Cytogenetics

50. The issue was discussed with…

The patient Patient’s mother Patient’s father

51. What is the suspected situation?

52. What is the type of the patient insurance?

Private insurance State insurance

Please write down any information or remark you would like to state.

Thank you very much for your contributions.

Res. Asst. Aslihan AKPINAR

Address: Kocaeli University, Medical Faculty, Department of Medical History and Ethics, 41380 Umuttepe – Izmit

Tel: 0 262 303 74 50

E-mail: [aslyakcay@yahoo.com](mailto:aslyakcay@yahoo.com)
